# Adaptive group bridge estimation for high-dimensional partially linear models

**DOI:** 10.1186/s13660-017-1432-x

**Published:** 2017-06-30

**Authors:** Xiuli Wang, Mingqiu Wang

**Affiliations:** School of Statistics, Qufu Normal University, Jingxuan West Road, Qufu, 273165 P.R. China

**Keywords:** 62E20, 62J07, 62F12, adaptive group bridge, high dimension, partially linear model

## Abstract

This paper studies group selection for the partially linear model with a diverging number of parameters. We propose an adaptive group bridge method and study the consistency, convergence rate and asymptotic distribution of the global adaptive group bridge estimator under regularity conditions. Simulation studies and a real example show the finite sample performance of our method.

## Introduction

Consider the following model:

1 $$ Y=\mathbf {x}^{T}\boldsymbol {\beta }+f(U)+\varepsilon, $$

where $\mathbf {x}=(\boldsymbol{x}_{1}^{T},\boldsymbol{x}_{2}^{T},\ldots,\boldsymbol{x}_{p_{n}}^{T})^{T}$ is a covariate vector with $\boldsymbol{x}_{j}=(X_{jk},k=1,\ldots,d_{j})^{T}$ being a $d_{j}\times1$ vector corresponding to the *j*th group in the linear part, $\boldsymbol {\beta }=(\boldsymbol {\beta }_{j}^{T},j=1,\ldots,p_{n})^{T}$ with $\boldsymbol {\beta }_{j}$ being the $d_{j}\times1$ vector of regression coefficients, *f* is an unknown function of *U*, and *ε* is the random error with mean zero. Without loss of generality, *U* is scaled to $[0, 1]$. Furthermore, $(\mathbf {x},U)$ and *ε* are independent.

Variable selection for high-dimensional data is a hot and important issue. Penalized regression methods have been widely used in the literature such as [1–5], and so on. Among these methods, bridge regression including lasso and ridge as two well-known special cases has been studied by many authors (e.g., [6–10]). [11] studied adaptive bridge estimation for high-dimensional linear models. In addition, group structure of variables arise always in many contemporary statistical modeling problems. [12] proposed a group bridge method which not only effectively removes unimportant groups, but also maintains the flexibility of selecting variables within identified groups. [13] investigated an adaptive choice of the penalty order in group bridge regression.

The aforementioned model (1) is just the partially linear model that originated from [14]. The partially linear model is a common semiparametric model enjoying the interpretability and flexibility. Our contributions in this paper include: (1) we propose an adaptive group bridge method to achieve the group selection for a high-dimensional partially linear model; (2) we consider the choice of index *γ* in the adaptive group bridge and use leave-one-observation-out cross-validation (CV) to implement this choice. It can significantly reduce the computational burden; (3) we give the consistency, convergence rate and asymptotic distribution of the adaptive group bridge estimator which is the global minimizer of the objective function.

The rest of the article is organized as follows. Section 2 gives the adaptive group bridge method. In Section 3, we show the assumptions and asymptotic results for the global adaptive group bridge estimator. Section 4 shows computational algorithm and selection of tuning parameters. Simulation studies and real data are presented in Section 5. Section 6 gives a short discussion. Technical proofs are relegated to Appendix.

## Adaptive group bridge in the partially linear model

Suppose that we have a collection of independent observations $\{(\mathbf {x}_{i},U_{i},Y_{i}), 1\leq i \leq n \}$ from model (1). That is,

2 $$ Y_{i} = \mathbf {x}^{T}_{i}\boldsymbol {\beta }+ f(U_{i}) + \varepsilon_{i},\quad i = 1, \ldots, n, $$

where $\varepsilon_{1}, \ldots, \varepsilon_{n}$ are i.i.d. random errors with mean zero and finite variance $\sigma^{2}<\infty$.

To obtain an estimate of function $f(\cdot)$, we employ a B-spline basis. Denote $\mathcal{S}_{n}$ as the space of polynomial splines of degree $m\geq 1$. Let $\{B_{k}(u), 1\leq k\leq q_{n}\}$ be a normalized B-spline basis with $\|B_{k}\|_{\infty}\leq1$, where $\|\cdot\|_{\infty}$ is the sup norm. Then, for any $f_{n}\in\mathcal{S}_{n}$, we have

$$f_{n}(u)=\sum_{j=1}^{q_{n}}B_{j}(u) \alpha_{j}\triangleq \mathbf {B}(u)^{T}\boldsymbol {\alpha }. $$

Under some smoothness conditions, the nonparametric function *f* can well be approximated by functions in $\mathcal{S}_{n}$.

Consider the following adaptive group bridge penalized objective function:

3 $$ \sum_{i=1}^{n} \bigl(Y_{i}-\mathbf {x}_{i}^{T}\boldsymbol {\beta }- \mathbf {B}(U_{i})^{T} \boldsymbol {\alpha }\bigr)^{2}+ \sum_{j=1}^{p_{n}} \lambda_{j}\|\boldsymbol {\beta }_{j}\|^{\gamma}, $$

where $\lambda_{j}$, $j=1,\ldots,p_{n}$, are the tuning parameters, and $\| \cdot\|$ denotes the $L_{2}$ norm on the Euclidean space. Let $\mathbf {Y}=(Y_{1},\ldots,Y_{n})^{T}$, $\mathbb {X}=(X_{ijk},1\leq i\leq n,1\leq j\leq p_{n}, 1\leq k\leq d_{j})=(\mathbf {x}_{1},\ldots, \mathbf {x}_{n})^{T}$ and $\mathbf {Z}=(\mathbf {B}(U_{1}),\ldots, \mathbf {B}(U_{n}))^{T}$. Then (3) can be changed into

4 $$ L_{n}(\boldsymbol {\beta },\boldsymbol {\alpha })=\|\mathbf {Y}-\mathbb {X}\boldsymbol {\beta }-\mathbf {Z}\boldsymbol {\alpha }\|^{2}+ \sum_{j=1}^{p_{n}}\lambda_{j}\| \boldsymbol {\beta }_{j}\|^{\gamma}. $$

For some ***β***, the optimal ***α*** minimizing $L_{n}(\cdot)$ meets the partial differential equation

$$\partial L_{n}(\boldsymbol {\beta },\boldsymbol {\alpha })/\partial \boldsymbol {\alpha }=0, $$

namely,

$$\mathbf {Z}^{T}\mathbf {Z}\boldsymbol {\alpha }=\mathbf {Z}^{T}(\mathbf {Y}-\mathbb {X}\boldsymbol {\beta }). $$

Let $H=\mathbf {Z}(\mathbf {Z}^{T}\mathbf {Z})^{-1}\mathbf {Z}^{T}$, note that *H* is a projection matrix. We can rewrite the expression (4) as follows:

5 $$ Q_{n}(\boldsymbol {\beta })= \bigl\Vert (I-H) (\mathbf {Y}-\mathbb {X}\boldsymbol {\beta }) \bigr\Vert ^{2} + \sum_{j=1}^{p_{n}} \lambda_{j} \Vert \boldsymbol {\beta }_{j} \Vert ^{\gamma}. $$

For some fixed $\gamma>0$, define $\hat {\boldsymbol {\beta }}=\arg\min Q_{n}(\boldsymbol {\beta })$, then $\hat {\boldsymbol {\beta }}$ is called the adaptive group bridge estimator. If $\hat {\boldsymbol {\beta }}$ is obtained, then the estimator $\hat {\boldsymbol {\alpha }}$ can be achieved. Thus we can get the estimator of the nonparametric part, namely, $\hat{f}_{n}(u)=\mathbf {B}(u)^{T}\hat {\boldsymbol {\alpha }}$.

## Asymptotic properties

In this section, we show the oracle property of the parametric part. For convenience of the statement, we first give some notations. Define $\mathbf{g}(u)=E(\mathbf {x}|U=u)$ and $\tilde {\mathbf {x}}=\mathbf {x}-E(\mathbf {x}|U)$. Let $\Sigma(u)$ be the conditional covariance matrix of $\tilde {\mathbf {x}}$, i.e., $\Sigma(u)=\operatorname{cov}(\tilde {\mathbf {x}}|U=u)$. Denote Ω as the unconditional covariance matrix of $\tilde {\mathbf {x}}$, i.e., $\Omega=E[\Sigma(U)]$. The corresponding sample version is $\mathbf {G}=(\mathbf{g}(U_{1}),\ldots,\mathbf{g}(U_{n}))^{T}$ with $\mathbf {g}(U_{i})=E(\mathbf {x}_{i}|U_{i})$ and $\widetilde {\mathbb {X}}=(\tilde {\mathbf {x}}_{1},\ldots, \tilde {\mathbf {x}}_{n})^{T}$ with $\tilde {\mathbf {x}}_{i}=\mathbf {x}_{i}-E(\mathbf {x}_{i}|U_{i})$.

Let the true parameter be $\boldsymbol {\beta }_{0}=(\boldsymbol {\beta }_{01}^{T},\ldots, \boldsymbol {\beta }_{0p_{n}}^{T})^{T}\triangleq(\boldsymbol {\beta }_{10}^{T},\boldsymbol {\beta }_{20}^{T})^{T}$. Let $\mathcal {A}= \{ 1\leq j \leq p_{n}: \|\boldsymbol {\beta }_{0j}\| \neq0\}$ be the index set of the nonzero groups. Without loss of generality, we assume that coefficients of the first $k_{n}$ group are nonzero, i.e., $\mathcal {A}=\{1,2,\ldots,k_{n}\}$. Let $|\mathcal {A}| = k_{n}$ be the cardinality of the set $\mathcal {A}$, which is allowed to increase with *n*. For $j\notin \mathcal {A}$, $\|\boldsymbol {\beta }_{0j}\|=0$. Define $\boldsymbol {\beta }_{10}=(\boldsymbol {\beta }_{0j}^{T},j\in \mathcal {A})^{T}$, $\boldsymbol {\beta }_{20}=(\boldsymbol {\beta }_{0j}^{T},j\notin \mathcal {A})^{T}$. Let $d^{*}=\max_{1\leq j \leq p_{n}}d_{j}$, $\varphi_{n1}=\max\{\lambda_{j},j\in \mathcal {A}\}$ and $\varphi_{n2}=\min\{ \lambda_{j},j\notin \mathcal {A}\}$.

Corresponding to the partition of $\boldsymbol {\beta }_{0}$, denote $\hat {\boldsymbol {\beta }}=(\hat {\boldsymbol {\beta }}_{(1)}^{T}, \hat {\boldsymbol {\beta }}_{(2)}^{T})^{T}$ and decompose

$$\mathbb {X}=(\mathbb {X}_{1} \mathbb {X}_{2}),\qquad \mathbf {G}=(\mathbf {G}_{1} \mathbf {G}_{2}),\qquad \widetilde {\mathbb {X}}=(\widetilde {\mathbb {X}}_{1} \widetilde {\mathbb {X}}_{2}), \qquad \Omega= \left ( \textstyle\begin{array}{@{}c@{\quad}c@{}}\Omega_{11} & \Omega_{12} \\ \Omega_{21} & \Omega_{22} \end{array}\displaystyle \right ). $$

The following conditions are required for the B-spline approximation of function *f*.

1. The distribution of *U* is absolutely continuous, and its density is bounded away from 0 and ∞.
2. (Hölder conditions of $f(\cdot)$ and $g_{j}(\cdot)$, where $g_{j}$ is the *j*th component of **g**) Let *l*, *δ* and *M* be real constants such that $0<\delta\leq1$ and $M>0$. $f(\cdot)$ and $g_{j}(\cdot)$ belong to a class of functions $\mathcal{H}$,
  $$ \mathcal{H}=\bigl\{ h: \bigl\vert h^{(l)}(u_{1})-h^{(l)}(u_{2}) \bigr\vert \leq M \vert u_{1}-u_{2} \vert ^{\delta }, \text{for } 0\leq u_{1}, u_{2} \leq1\bigr\} , $$
  where $0< l\leq m-1$ and $r=l+\delta$.

The following part lists all the reasonable conditions which are necessary to attain the asymptotic results.

Let $\lambda_{\max}(\Omega)$ and $\lambda_{\min }(\Omega)$ be the largest and smallest eigenvalue of Ω, respectively. There exist constants $\tau_{1}$ and $\tau_{2}$ such that
  $$0< \tau_{1}\leq\lambda_{\min}(\Omega)\leq\lambda_{\max}( \Omega )\leq\tau_{2}< \infty. $$
There exist constants $0< b_{0}< b_{1}<\infty$ such that
  $$b_{0}\leq\min\bigl\{ \Vert \boldsymbol {\beta }_{0j} \Vert , 1\leq j \leq k_{n}\bigr\} \leq\max\bigl\{ \Vert \boldsymbol {\beta }_{0j} \Vert , 1\leq j\leq k_{n}\bigr\} \leq b_{1}. $$
$\|n^{-1}\mathbb {X}^{T}(I-H)\mathbb {X}-\Omega\|\stackrel{P}{\rightarrow}0$; $E[\operatorname{tr}(\mathbb {X}^{T}(I-H)\mathbb {X})]=O(np_{n})$.
$d^{*}=O(1)$, $p_{n}^{2}/n\rightarrow0$ and $n^{-1}\varphi _{n1}k_{n}\rightarrow0$.
(a) $\varphi_{n1}k_{n}^{1/2}/(\sqrt{np_{n}}+n\sqrt {p_{n}}q_{n}^{-r})\rightarrow0$; (b) $\varphi_{n2}(\sqrt{n^{-1}p_{n}}+\sqrt {p_{n}}q_{n}^{-r})^{\gamma-2}/n\rightarrow\infty$.
For every $1\leq j \leq p_{n}$ and $1\leq k\leq d_{j}$, $E[X_{1jk}-E(X_{1jk}|U_{1})]^{4}$ is bounded. Furthermore, $E(\varepsilon ^{4})$ is bounded.

Conditions (A1) and (A2) are commonly used. Condition (A3) holds under some conditions. The proof can be found in Lemmas 1 and 2 in [15]. Condition (A4) is used to obtain the consistency of the estimator. Condition (A5) is needed in the proof of convergence rate. Condition (A6) is necessary to attain the asymptotic distribution.

### Theorem 3.1

Consistency

*Suppose that* $\gamma>0$ *and conditions* (A1)-(A4) *hold*, *then*

$$\Vert \hat {\boldsymbol {\beta }}-\boldsymbol {\beta }_{0} \Vert ^{2}=O_{P} \bigl(n^{-1}d^{*}p_{n}+q_{n}^{-2r}+n^{-1} \varphi_{n1}k_{n}\bigr), $$

*namely*, $\|\hat {\boldsymbol {\beta }}-\boldsymbol {\beta }_{0}\|\stackrel{P}{\rightarrow}0$.

Theorem 3.1 implies that under some conditions the estimators converge to the true values of parameters.

### Theorem 3.2

Convergence rate

*Suppose that conditions* (A1)-(A5) *hold*, *then*

$$\Vert \hat {\boldsymbol {\beta }}-\boldsymbol {\beta }_{0} \Vert =O_{P}\bigl( \sqrt{n^{-1}p_{n}}+\sqrt{p_{n}}q_{n}^{-r} \bigr). $$

This theorem shows that the adaptive group bridge can give the optimal convergence rate with $p_{n}\rightarrow\infty$.

### Theorem 3.3

Oracle property

*Suppose that* $0<\gamma<1$, $n^{-1}k_{n}q_{n}\rightarrow0$ *and* $nq_{n}^{-2r}\rightarrow0$. *If conditions* (A1)-(A6) *are satisfied*, *then we have*

(i) $\Pr(\hat {\boldsymbol {\beta }}_{(2)}={\mathbf{0}})\rightarrow1$, $n\rightarrow\infty$;
(ii) *Let* $u_{n}^{2}=n^{2}\boldsymbol {\omega }_{n}^{T}(\mathbb {X}^{T}_{1}(I-H)\mathbb {X}_{1})^{-1}\Omega _{11}(\mathbb {X}^{T}_{1}(I-H)\mathbb {X}_{1})^{-1}\boldsymbol {\omega }_{n}$ *with* $\boldsymbol {\omega }_{n}$ *being some* $\sum_{j=1}^{k_{n}}d_{j}$-*vector with* $\|\boldsymbol {\omega }_{n}\|^{2}=1$, *then*
  $$n^{1/2}u_{n}^{-1}\boldsymbol {\omega }_{n}^{T}( \hat {\boldsymbol {\beta }}_{(1)}-\boldsymbol {\beta }_{10})\stackrel{D}{\rightarrow }N\bigl(0, \sigma^{2}\bigr). $$

This theorem states that the adaptive group bridge performs as well as the oracle [16].

## Computational algorithm and selection of tuning parameters

### Computational algorithm

In this section, we apply the LQA algorithm proposed by [3] to compute the adaptive group bridge estimate.

We take the initial value ${\boldsymbol {\beta }}^{(0)}$. Here the ordinary least square estimate is chosen as the initial value $\boldsymbol {\beta }^{(0)}$. The penalty term $p_{\lambda_{j}}(\|{\boldsymbol {\beta }}_{j}\|)=\lambda_{j}\| \boldsymbol {\beta }_{j}\|^{\gamma}$ can be approximated as

$$p_{\lambda_{j}}\bigl( \Vert {\boldsymbol {\beta }}_{j} \Vert \bigr)\approx p_{\lambda_{j}}\bigl( \bigl\Vert {\boldsymbol {\beta }}_{j}^{(0)} \bigr\Vert \bigr)+\frac{1}{2}\bigl\{ p_{\lambda_{j}}'\bigl( \bigl\Vert {\boldsymbol {\beta }}_{j}^{(0)} \bigr\Vert \bigr)/ \bigl\Vert { \boldsymbol {\beta }}_{j}^{(0)} \bigr\Vert \bigr\} \bigl( \Vert { \boldsymbol {\beta }}_{j} \Vert ^{2}- \bigl\Vert {\boldsymbol {\beta }}_{j}^{(0)} \bigr\Vert ^{2}\bigr), $$

when $\|{\boldsymbol {\beta }}_{j}^{(0)}\|>0$. The following iterative expression of ***β*** can be obtained:

6 $$\begin{aligned} \boldsymbol {\beta }^{(1)}=\bigl[\mathbb {X}^{T}(I-H)\mathbb {X}+n \Sigma_{\lambda,\gamma}\bigl({\boldsymbol {\beta }}^{(0)}\bigr)\bigr]^{-1} \mathbb {X}^{T}(I-H)\mathbf {Y}, \end{aligned}$$

where

$$\begin{aligned} \Sigma_{\lambda,\gamma}\bigl({\boldsymbol {\beta }}^{(0)}\bigr)=\operatorname{diag} \biggl\{ \frac {p_{\lambda_{j}}'(\|{\boldsymbol {\beta }}^{(0)}_{j}\|)}{\|{\boldsymbol {\beta }}^{(0)}_{j}\| }I_{d_{j}},j=1,\ldots,p_{n} \biggr\} , \end{aligned}$$

with $I_{d_{j}}$ being a $d_{j}\times d_{j}$ unit matrix. If some $\|\boldsymbol {\beta }^{(1)}_{j}\|$ is smaller than 10^−3^, then we set $\boldsymbol {\beta }^{(1)}_{j}={\mathbf{0}}$. The finial estimate can be obtained iteratively by formula (6) until the convergence is achieved.

### Selection of the tuning parameters

For our method, $q_{n}$, *γ*, and $\lambda_{j}$ ($j=1,\ldots,p_{n}$) should be chosen. For convenience, cubic spline basis ($m=4$) is used. We set $q_{n}=7$. Simulation results demonstrate that this choice performs quite well. There are also many tuning parameters that should be chosen. In fact, we only need to select one tuning parameter by setting $\lambda_{j}=\lambda/\|\boldsymbol {\beta }_{j}^{(0)}\|$. We use ‘leave-one-observation-out’ cross-validation (CV) to select *λ* and *γ*. Due to the convergence of the algorithm, we have

$$\hat {\boldsymbol {\beta }}=\bigl[\mathbb {X}^{T}(I-H)\mathbb {X}+n\Sigma_{\lambda,\gamma}({\hat {\boldsymbol {\beta }}}) \bigr]^{-1}\mathbb {X}^{T}(I-H)\mathbf {Y}, $$

where $\hat {\boldsymbol {\beta }}$ is obtained based on the whole data set. Note that it is the solution of the ridge regression

7 $$ \bigl\Vert \mathbf {Y}^{*}-\mathbb {X}^{*}\boldsymbol {\beta }\bigr\Vert ^{2}+n \boldsymbol {\beta }^{T}\Sigma_{\lambda,\gamma}({\hat {\boldsymbol {\beta }}})\boldsymbol {\beta }, $$

where $\mathbf {Y}^{*}=(I-H)\mathbf {Y}$ and $\mathbb {X}^{*}=(I-H)\mathbb {X}$. Let $\mathbf {Y}^{*}=(y_{1}^{*},\ldots,y_{n}^{*})^{T}$ and $\mathbb {X}^{*}=(\mathbf {x}_{1}^{*},\ldots, \mathbf {x}_{n}^{*})^{T}$. The CV error is

$$CV(\lambda,\gamma)=\frac{1}{n}\sum_{i=1}^{n} \bigl(y_{i}^{*}-\mathbf {x}_{i}^{*T}\hat {\boldsymbol {\beta }}^{-i} \bigr)^{2}, $$

where $\hat {\boldsymbol {\beta }}^{-i}$ is achieved by solving (7) without the *i*th observation. The computation of the CV error is intensive, so we will use the following formula, which can be proved similar to [17]:

$$CV(\lambda,\gamma)=\frac{1}{n}\sum_{i=1}^{n} \bigl(y_{i}^{*}-\mathbf {x}_{i}^{*T}\hat {\boldsymbol {\beta }}\bigr)^{2}/(1-D_{ii}), $$

where $D_{ii}$ is the $(i,i)$th diagonal element of $(I-H)\mathbb {X}[\mathbb {X}^{T}(I-H)\mathbb {X}+n\Sigma_{\lambda,\gamma}({\hat {\boldsymbol {\beta }}})]^{-1}\mathbb {X}^{T}(I-H)$. It is obvious that this method can significantly reduce the computational burden.

## Simulation studies and application

In this section, we investigate the finite sample performance of the adaptive group bridge method through simulations and a real data application.

### Monte Carlo simulations

We simulate 100 datasets consisting of *n* observations from the following partially linear model:

$$ Y_{i}=\sum_{j=1}^{p_{n}} \boldsymbol{x}_{ij}^{T}\boldsymbol {\beta }_{j}+\cos(2\pi U_{i})+\varepsilon _{i},\quad i=1,\ldots,n, $$

where $n=500$, and the error $\varepsilon_{i}\sim N(0,\sigma^{2})$ with $\sigma=0.5,1,4$. We consider that there are $p_{n}$ groups with $p_{n}=10,30,50$ and each group consists of three variables. The true values of parameters $\boldsymbol {\beta }_{1}^{T}=(0.5,1,1.5)$, $\boldsymbol {\beta }_{2}^{T}=(1,-1,1)$, $\boldsymbol {\beta }_{3}^{T}=(0.5,0.5,0.5)$, $\boldsymbol {\beta }_{4}^{T}=\cdots =\boldsymbol {\beta }_{p_{n}}^{T}=(0,0,0)$. $U_{i}$ follows the uniform distribution on $[0,1]$. To generate covariate $\mathbf {x}=(\boldsymbol{x}_{1}^{T},\boldsymbol{x}_{2}^{T},\ldots,\boldsymbol{x}_{p_{n}}^{T})^{T}$ with $\boldsymbol{x}_{j}=(X_{jk},k=1,2,3)^{T}$, we first simulate $R_{1},\ldots ,R_{3p_{n}}$ independently from the standard normal distribution. Next, simulate $Z_{j}$, $j=1,\ldots,p_{n}$, from a multivariate normal distribution with the mean zero and $\operatorname{Cov}(Z_{j},Z_{l})=0.6^{|j-l|}$. Then the covariates are generated as $X_{jk}=(Z_{j}+R_{3(j-1)+k})/\sqrt{2}$, $j=1,\ldots,p_{n}$, $k=1,2,3$.

We compare the adaptive group bridge (AGB) with the group lasso (GL) and the group bridge (GB). The following three performance measures are calculated:

1. $L_{2}$ loss of parametric estimate, which is defined as $\|\widehat {\boldsymbol {\beta }}-\boldsymbol {\beta }_{0}\|$.
2. Average number of nonzero groups identified by the method (NN).
3. Average number of nonzero groups identified by the method that are truly nonzero (NNT).

**Figure 1:**
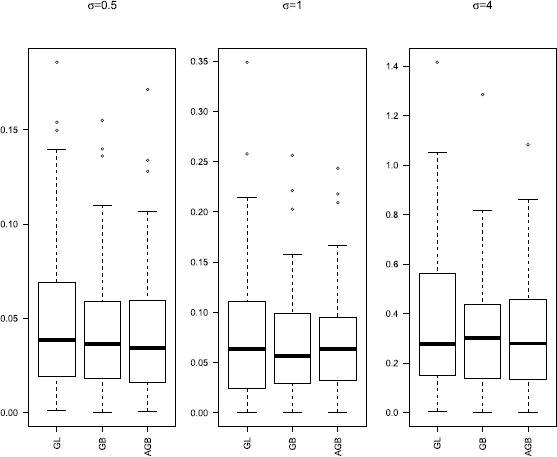
**Boxplots of** $\pmb{L_{2}}$ **loss for** $\pmb{p_{n}=10}$ **.**

**Figure 2:**
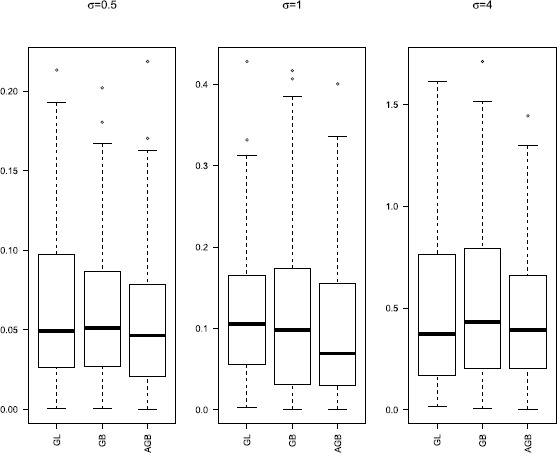
**Boxplots of** $\pmb{L_{2}}$ **loss for** $\pmb{p_{n}=30}$ **.**

**Figure 3:**
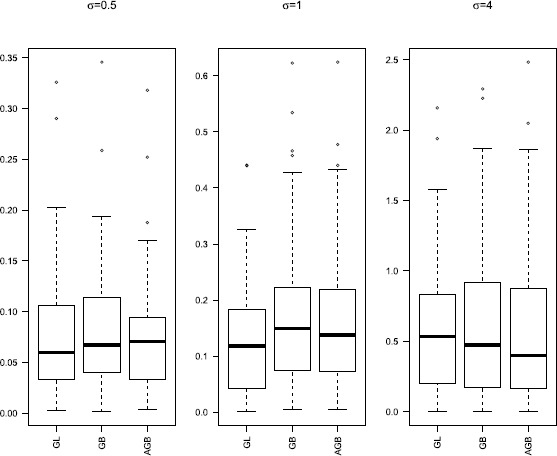
**Boxplots of** $\pmb{L_{2}}$ **loss for** $\pmb{p_{n}=50}$ **.**

Group selection results are depicted in Table 1. The numbers in the parentheses in the columns labeled ‘NN’ and ‘NNT’ are the corresponding sample standard deviations based on the 100 runs. Boxplots of the $L_{2}$ losses under different settings are given in Figures 1-3.

**Table 1:** **Group selection results**

$\boldsymbol{p_{n}}$	**Method**	***σ*** ** = 0.5**		***σ*** ** = 1**		***σ*** ** = 4**	
		**NN**	**NNT**	**NN**	**NNT**	**NN**	**NNT**
10	GL	7.80	3	7.59	3	6.02	3
		(1.231)	(0)	(2.396)	(0)	(1.255)	(0)
	GB	4.64	3	4.80	3	4.55	3
		(1.259)	(0)	(1.356)	(0)	(1.258)	(0)
	AGB	5.22	3	5.00	3	4.56	3
		(1.605)	(0)	(1.735)	(0)	(1.131)	(0)
30	GL	20.47	3	11.49	3	14.46	3
		(2.504)	(0)	(4.464)	(0)	(2.022)	(0)
	GB	11.04	3	10.96	3	10.18	3
		(3.643)	(0)	(3.784)	(0)	(2.350)	(0)
	AGB	13.06	3	10.64	3	9.57	3
		(5.510)	(0)	(5.921)	(0)	(2.046)	(0)
50	GL	33.17	3	16.48	3	22.37	3
		(3.223)	(0)	(5.018)	(0)	(3.308)	(0)
	GB	17.23	3	17.79	3	15.96	3
		(4.608)	(0)	(3.952)	(0)	(3.284)	(0)
	AGB	19.25	3	15.67	3	15.68	3
		(8.437)	(0)	(8.385)	(0)	(3.684)	(0)

From Table 1, we can have the following observations:

1. Both GB and AGB perform better than GL for all settings. All these three methods can retain all the true nonzero groups, but GL always keeps more redundant groups that are unrelated with the response than both GB and AGB.
2. AGB performs much better for larger *σ* and $p_{n}$. When $p_{n}=50$ for AGB, groups selected for the case $\sigma=4$ are about 18.5% lower than that for the case $\sigma=0.5$. While groups selected for GB decrease by 7.37% in the same situation.
3. For $p_{n}=10$, GB performs better than AGB, but the stability of GB is bad for $\sigma=4$.

Figures 1-3 present $L_{2}$ losses with varying *σ* and $p_{n}$. We can see that the performances of estimates are similar for GB and AGB. For $p_{n}=30$ and 50, both GB and AGB perform better than GL. However, when $p_{n}=50$, the median of $L_{2}$ losses for all these three are similar for $\sigma =0.5\mbox{ and }4$, but the $L_{2}$ losses of GL fluctuate more widely.

### Wage data analysis

The workers’ wage data from Berndt[18] contains a random sample of 534 observations on 11 variables sampled from the current population survey of 1985. It provides information on wages and other characteristics of the workers, including continuous variables: the number of years of education, years of work experience, age and nominal variables: race, sex, region of residence, occupational status, sector, marital status and union membership. Our goal is to study the important factors for the wage, so it is reasonable to use our proposed method for these data.

From the residual plot, we can easily see that the variance of wages is not a constant. So the *log* transformation is used to stabilize the variance of wages. Due to the multicollinearity problem between age and experience, we need to get rid of either age or experience. Here we remove the age variable from the model. Xie and Huang [15] analyzed these data without considering the transformation of *Y*. Furthermore, they did not consider group selection of factors. Similar to Xie and Huang [15], we fit these data using a partially linear model with *U* being ‘years of work experience’.

Table 2 reports estimated regression coefficients of GL, GB and AGB. All these three methods exclude marital status. We use the first 400 observations as a training dataset to select and fit the model, and use the rest of 134 observations as a testing dataset to evaluate the prediction ability of the selected model. The prediction performance is measured by the median of $\{|y_{i}-\hat{y}_{i}|, i = 1, 2, \ldots, 134\} $ for GL, GB and AGB using the testing data, respectively. Here $y_{i}$’s are those 134 observations in the testing dataset and $\hat{y}_{i}$’s are corresponding prediction values. The median absolute prediction errors of GL, GB and AGB are 0.3072, 0.3062 and 0.3022, respectively. Therefore, we can conclude that the AGB gives the smallest prediction error, so it is an attractive technique in group selection.

**Table 2:** **Estimates of the wage data**

**Variable**	**Description**	**GL**	**GB**	**AGB**
edu	Number of years of education	0.0694	0.0668	0.0635
south	1 = southern region, 0 = other	−0.0723	−0.0679	−0.0490
sex	1 = Female, 0 = Male	−0.1999	−0.1983	−0.2031
union	1 = union member, 0 = nonmember	0.1951	0.1934	0.2030
race	1 = other, 0 = White	−0.0559	−0.0585	−0.0582
	1 = Hispanic, 0 = White	−0.0537	−0.0615	−0.0614
occup	1 = management, 0 = other	0.1874	0.2173	0.2516
	1 = sales, 0 = other	−0.0797	−0.0809	−0.0721
	1 = clerical, 0 = other	0.0166	0.0262	0.0430
	1 = service, 0 = other	−0.1171	−0.1173	−0.1104
	1 = professional, 0 = other	0.1533	0.1768	0.2061
sector	1 = manufacturing, 0 = other	0.0848	0.0912	0.0994
	1 = construction, 0 = other	0.0546	0.0622	0.0674
marr	1 = married, 0 = other	0.0000	0.0000	0.0000

## Discussion

This paper studies group selection for high-dimensional partially linear model with the adaptive group bridge method. We also consider the choice of *γ* in the bridge penalty. It is worth mentioning that we use ‘leave-one-observation-out’ cross-validation to select both *λ* and *γ*. This method can significantly reduce the computational burden. This is the first try to use this method in group selection for the partially linear model.

## Appendix

### Proof of Theorem 3.1

By the definition of $\hat {\boldsymbol {\beta }}$, it is easy to get

$$\bigl\Vert (I-H) (\mathbf {Y}-\mathbb {X}\hat {\boldsymbol {\beta }}) \bigr\Vert ^{2}+ \sum _{j=1}^{p_{n}}\lambda_{j} \Vert \hat {\boldsymbol {\beta }}_{j} \Vert ^{\gamma}\leq \bigl\Vert (I-H) (\mathbf {Y}-\mathbb {X}\boldsymbol {\beta }_{0}) \bigr\Vert ^{2}+ \sum _{j=1}^{p_{n}}\lambda_{j} \Vert \boldsymbol {\beta }_{0j} \Vert ^{\gamma}, $$

that is,

$$\bigl\Vert (I-H) (\mathbf {Y}-\mathbb {X}\hat {\boldsymbol {\beta }}) \bigr\Vert ^{2}- \bigl\Vert (I-H) (\mathbf {Y}-\mathbb {X}\boldsymbol {\beta }_{0}) \bigr\Vert ^{2}\leq \sum _{j=1}^{p_{n}}\lambda_{j} \Vert \boldsymbol {\beta }_{0j} \Vert ^{\gamma}. $$

As $\mathbf {Y}= \mathbb {X}\boldsymbol {\beta }_{0} + \boldsymbol {f}(\mathbf{U}) + {\boldsymbol{\varepsilon}}$ with $\boldsymbol {f}(\mathbf{U})=(f(U_{1}),\ldots,f(U_{n}))^{T}$ and ${\boldsymbol{\varepsilon}}=(\varepsilon_{1},\ldots,\varepsilon_{n})^{T}$, we can rewrite the upper inequality as follows:

$$\bigl\Vert (I-H)\mathbb {X}(\hat {\boldsymbol {\beta }}-\boldsymbol {\beta }_{0}) \bigr\Vert ^{2}-2 \bigl(\boldsymbol {f}(\mathbf{U}) + {\boldsymbol{\varepsilon}}\bigr)^{T}(I-H)\mathbb {X}( \hat {\boldsymbol {\beta }}-\boldsymbol {\beta }_{0})\leq \sum_{j=1}^{p_{n}} \lambda_{j} \Vert \boldsymbol {\beta }_{0j} \Vert ^{\gamma}. $$

Let

$$\begin{aligned}& a_{n}=n^{-1/2}\bigl[\mathbb {X}^{T}(I-H)\mathbb {X}\bigr]^{1/2}(\hat {\boldsymbol {\beta }}-\boldsymbol {\beta }_{0}), \\& b_{n}=n^{-1/2}\bigl[\mathbb {X}^{T}(I-H)\mathbb {X}\bigr]^{-1/2}\mathbb {X}^{T}(I-H) \bigl(\boldsymbol {f}(\mathbf{U}) + {\boldsymbol {\varepsilon }}\bigr). \end{aligned}$$

Then we have

$$\Vert a_{n} \Vert ^{2}\leq2\bigl( \Vert a_{n}-b_{n} \Vert ^{2}+ \Vert b_{n} \Vert ^{2}\bigr)\leq\frac{2}{n}\sum _{j=1}^{p_{n}}\lambda_{j} \Vert \boldsymbol {\beta }_{0j} \Vert ^{\gamma}+4 \Vert b_{n} \Vert ^{2}. $$

Since $|\mathcal {A}|=k_{n}$, under condition (A2),

$$\frac{2}{n}\sum_{j=1}^{p_{n}} \lambda_{j}\|\boldsymbol {\beta }_{0j}\|^{\gamma}=O\biggl( \frac {\varphi_{n1} k_{n}}{n}\biggr). $$

While

8 $$\begin{aligned} \|b_{n}\|^{2} =&\frac{1}{n}\bigl(\boldsymbol {f}(\mathbf{U}) + \boldsymbol {\varepsilon }\bigr)^{T}(I-H)\mathbb {X}\bigl[\mathbb {X}^{T}(I-H)\mathbb {X}\bigr]^{-1}\mathbb {X}^{T}(I- H) \bigl(\boldsymbol {f}(\mathbf{U}) + \boldsymbol {\varepsilon }\bigr) \\ \leq&\frac{2}{n}\boldsymbol {\varepsilon }^{T} A\boldsymbol {\varepsilon }+\frac{2}{n}\boldsymbol {f}( \mathbf{U})^{T} A\boldsymbol {f}(\mathbf{U}), \end{aligned}$$

where

$$A=(I-H)\mathbb {X}\bigl[\mathbb {X}^{T}(I-H)\mathbb {X}\bigr]^{-1} \mathbb {X}^{T}(I- H). $$

For the first term on the right-hand side of (8),

$$ E \biggl(\frac{1}{n}\boldsymbol {\varepsilon }^{T}A\boldsymbol {\varepsilon }\biggr) = \frac{\sigma^{2}}{n} \operatorname{tr}\bigl(E(A)\bigr)\leq n^{-1}d^{*}p_{n} \sigma^{2}. $$

Thus

9 $$ n^{-1}\boldsymbol {\varepsilon }^{T} A\boldsymbol {\varepsilon }=O_{P} \bigl(n^{-1}d^{*}p_{n}\bigr). $$

For the second term on the right-hand side of (8), by conditions (C1) and (C2),

10 $$\begin{aligned} E \biggl(\frac{1}{n}\boldsymbol {f}(\mathbf{U})^{T} A\boldsymbol {f}( \mathbf{U}) \biggr) \leq&\frac{1}{n}E\bigl\{ \lambda_{\max}\bigl\{ (I-H)\mathbb {X}\bigl[\mathbb {X}^{T}(I- H)\mathbb {X}\bigr]^{-1} \mathbb {X}^{T}(I-H)\bigr\} \\ &{}\times \operatorname{tr}\bigl[\boldsymbol {f}(\mathbf{U})^{T}(I-H)\boldsymbol {f}( \mathbf {U})\bigr]\bigr\} \\ =&\frac{1}{n}E\bigl[\boldsymbol {f}(\mathbf{U})^{T}(I-H)\boldsymbol {f}(\mathbf{U}) \bigr] =O\bigl(q_{n}^{-2r}\bigr). \end{aligned}$$

Combining (9)-(10),

$$\|b_{n}\|^{2}=O_{P}\bigl(n^{-1}d^{*}p_{n}+q_{n}^{-2r} \bigr). $$

By conditions (A1) and (A3),

$$\begin{aligned} E\|a_{n}\|^{2} =&\frac{1}{n}E\bigl[(\hat {\boldsymbol {\beta }}- \boldsymbol {\beta }_{0})^{T}\mathbb {X}^{T}(I-H)\mathbb {X}(\hat {\boldsymbol {\beta }}-\boldsymbol {\beta }_{0})\bigr] \\ =&E \biggl[(\hat {\boldsymbol {\beta }}-\boldsymbol {\beta }_{0})^{T} \biggl(\frac{1}{n} \mathbb {X}^{T}(I-H)\mathbb {X}-\Omega \biggr) (\hat {\boldsymbol {\beta }}-\boldsymbol {\beta }_{0}) \biggr] \\ &{}+E\bigl[(\hat {\boldsymbol {\beta }}-\boldsymbol {\beta }_{0})^{T} \Omega(\hat {\boldsymbol {\beta }}- \boldsymbol {\beta }_{0})\bigr] \\ \geq&\frac{\tau_{1}}{2}E\|\hat {\boldsymbol {\beta }}-\boldsymbol {\beta }_{0}\|^{2}. \end{aligned}$$

Therefore

$$\|\hat {\boldsymbol {\beta }}-\boldsymbol {\beta }_{0}\|^{2}=O_{P}\bigl(n^{-1}d^{*}p_{n}+q_{n}^{-2r}+n^{-1} \varphi_{n1}k_{n}\bigr). $$

Under condition (A4), we have

$$\|\hat {\boldsymbol {\beta }}-\boldsymbol {\beta }_{0}\|\stackrel{P}{\rightarrow}0. $$

 □

### Proof of Theorem 3.2

Let $\mu_{n}=\sqrt{n^{-1}p_{n}}+q_{n}^{-r}+\sqrt{n^{-1}\varphi_{n1}k_{n}}$, we can choose a sequence $\{r_{n}, r_{n}>0\}$ which satisfies $r_{n}\rightarrow0$. Partition $\mathbb{R}^{\sum _{j=1}^{p_{n}}d_{j}}\backslash\{0\}$ into shells $\{S_{nj}: j=1,2,\ldots\}$, where $S_{nj}=\{\boldsymbol {\beta }: 2^{j-1}r_{n}\leq\|\boldsymbol {\beta }-\boldsymbol {\beta }_{0}\|<2^{j}r_{n}\}$. For an arbitrary fixed constant $L\in\mathbb{R}^{+}$, if $\|\hat {\boldsymbol {\beta }}-\boldsymbol {\beta }_{0}\|$ is larger than $2^{L}r_{n}$, $\hat {\boldsymbol {\beta }}$ is in one of the shells with $j\geq L$, we have

$$\begin{aligned} \Pr\bigl( \Vert \hat {\boldsymbol {\beta }}-\boldsymbol {\beta }_{0} \Vert \geq2^{L}r_{n} \bigr) =&\sum_{l>L,2^{l}r_{n}>2^{L_{1}}\mu _{n}}\Pr(\hat {\boldsymbol {\beta }}\in S_{nl}) \\ &{}+\sum_{l>L,2^{l}r_{n}\leq2^{L_{1}}\mu_{n}}\Pr(\hat {\boldsymbol {\beta }}\in S_{nl}) \quad (L_{1}\mbox{ is an arbitrary constant}), \end{aligned}$$

where

$$ \sum_{l>L,2^{l}r_{n}>2^{L_{1}}\mu_{n}}\Pr(\hat {\boldsymbol {\beta }}\in S_{nl}) \leq\Pr\bigl( \|\hat {\boldsymbol {\beta }}-\boldsymbol {\beta }_{0}\|\geq2^{L_{1}-1}\mu_{n}\bigr)=o(1), $$

and

$$\begin{aligned}& \sum_{l>L,2^{l}r_{n}\leq2^{L_{1}}\mu_{n}}\Pr(\hat {\boldsymbol {\beta }}\in S_{nl}) \\& \quad =\sum_{l>L,2^{l}r_{n}\leq2^{L_{1}}\mu_{n}}\Pr \biggl(\hat {\boldsymbol {\beta }}\in S_{nl}, \|\Delta_{n}\|\leq\frac{\tau_{1}}{2} \biggr) \\& \qquad {}+\sum_{l>L,2^{l}r_{n}\leq2^{L_{1}}\mu_{n}}\Pr \biggl(\hat {\boldsymbol {\beta }}\in S_{nl},\|\Delta_{n}\|>\frac{\tau_{1}}{2} \biggr), \end{aligned}$$

where $\Delta_{n}=n^{-1}\mathbb {X}^{T}(I-H)\mathbb {X}-\Omega$. By condition (A3),

$$ \sum_{l>L,2^{l}r_{n}\leq2^{L_{1}}\mu_{n}}\Pr \biggl(\hat {\boldsymbol {\beta }}\in S_{nl},\| \Delta_{n}\|>\frac{\tau_{1}}{2} \biggr)\leq\Pr \biggl(\|\Delta _{n}\|>\frac{\tau_{1}}{2} \biggr)=o(1). $$

Therefore,

$$\begin{aligned}& \Pr\bigl(\|\hat {\boldsymbol {\beta }}-\boldsymbol {\beta }_{0}\|\geq2^{L}r_{n}\bigr) \\& \quad =o(1)+\sum_{l>L,2^{l}r_{n}\leq2^{L_{1}}\mu_{n}}\Pr \biggl(\inf _{\boldsymbol {\beta }\in S_{nl}}\bigl(Q_{n}(\boldsymbol {\beta })-Q_{n}( \boldsymbol {\beta }_{0})\bigr)< 0,\|\Delta_{n}\|\leq\frac{\tau _{1}}{2} \biggr). \end{aligned}$$

Since

$$\begin{aligned}& Q_{n}(\boldsymbol {\beta })-Q_{n}(\boldsymbol {\beta }_{0}) \\& \quad = \bigl\Vert (I-H)\mathbb {X}(\boldsymbol {\beta }-\boldsymbol {\beta }_{0}) \bigr\Vert ^{2}-2\bigl(\boldsymbol {f}(\mathbf{U}) +\boldsymbol {\varepsilon }\bigr)^{T}(I-H)\mathbb {X}(\boldsymbol {\beta }- \boldsymbol {\beta }_{0}) \\& \qquad {}+\sum_{j=1}^{p_{n}} \lambda_{j}\bigl( \Vert \boldsymbol {\beta }_{j} \Vert ^{\gamma}- \Vert \boldsymbol {\beta }_{0j} \Vert ^{\gamma}\bigr) \\& \quad \geq \bigl\Vert (I-H)\mathbb {X}(\boldsymbol {\beta }-\boldsymbol {\beta }_{0}) \bigr\Vert ^{2}-2\bigl(\boldsymbol {f}(\mathbf{U}) +\boldsymbol {\varepsilon }\bigr)^{T}(I-H)\mathbb {X}(\boldsymbol {\beta }- \boldsymbol {\beta }_{0}) \\& \qquad {}+\sum_{j=1}^{k_{n}} \lambda_{j}\bigl( \Vert \boldsymbol {\beta }_{j} \Vert ^{\gamma}- \Vert \boldsymbol {\beta }_{0j} \Vert ^{\gamma}\bigr) \\& \quad \stackrel{\Delta}{=} {\mathrm{I}}_{n1}+{\mathrm{I}}_{n2}+{ \mathrm{I}}_{n3}. \end{aligned}$$

For ${\mathrm{I}}_{n1}$,

$$ {\mathrm{I}}_{n1} \geq \inf_{\boldsymbol {\beta }\in S_{nl}}\frac{n\tau_{1}}{2}\| \boldsymbol {\beta }-\boldsymbol {\beta }_{0}\|^{2}, $$

for all $\boldsymbol {\beta }\in S_{nl}$, there exists $\|\boldsymbol {\beta }-\boldsymbol {\beta }_{0}\|^{2}\geq2^{2l-2}r^{2}_{n}$, therefore ${\mathrm{I}}_{n1} \geq n\tau_{1} 2^{2l-3}r_{n}^{2}$.

For ${\mathrm{I}}_{n3}$, we have

$$\begin{aligned} \begin{aligned} |{\mathrm{I}}_{n3}| &=\sum_{j=1}^{k_{n}} \lambda_{j}\gamma \bigl\Vert \boldsymbol {\beta }_{j}^{*} \bigr\Vert ^{\gamma-1}\bigl( \Vert \boldsymbol {\beta }_{j} \Vert - \Vert \boldsymbol {\beta }_{0j} \Vert \bigr) \\ &\leq\varphi_{n1}\gamma\sum_{j=1}^{k_{n}} \bigl\Vert \boldsymbol {\beta }_{j}^{*} \bigr\Vert ^{\gamma -1} \Vert \boldsymbol {\beta }_{j}-\boldsymbol {\beta }_{0j} \Vert , \end{aligned} \end{aligned}$$

where $\boldsymbol {\beta }_{j}^{*}$ is between $\boldsymbol {\beta }_{j}$ and $\boldsymbol {\beta }_{0j}$. By condition (A2) and since we only need to consider ***β*** with $\boldsymbol {\beta }\in S_{nl}$, $2^{l}r_{n}\leq2^{L_{1}}\mu_{n}$, there exists a constant $C_{3} > 0$ such that

$$|{\mathrm{I}}_{n3}| \leq C_{3}\varphi_{n1}\gamma \sum_{j=1}^{k_{n}} \|\boldsymbol {\beta }_{j}- \boldsymbol {\beta }_{0j}\| \leq C_{3}\varphi_{n1}k_{n}^{1/2} \gamma\|\boldsymbol {\beta }-\boldsymbol {\beta }_{0}\|. $$

So for all $\boldsymbol {\beta }\in S_{nl}$ such that $|{{\mathrm{I}}}_{n3}|\leq C_{3}\varphi_{n1}k_{n}^{1/2}\gamma2^{l}r_{n}$, by the Markov inequality, we have

$$\begin{aligned}& \Pr \Bigl(\inf_{\boldsymbol {\beta }\in S_{nl}}\bigl({Q}_{n}( \boldsymbol {\beta })-{Q}_{n}(\boldsymbol {\beta }_{0})\bigr)\leq 0 \Bigr) \\& \quad \leq\Pr \Bigl(\sup_{\boldsymbol {\beta }\in S_{nl}}|{\mathrm{I}}_{n2}| \geq n\tau_{1} 2^{2l-3}r^{2}_{n}-C_{3} \varphi_{n1}k_{n}^{1/2}\gamma2^{l}r_{n} \Bigr) \\& \quad \leq\frac{E (\sup_{\boldsymbol {\beta }\in S_{nl}}|{{\mathrm{I}}}_{n2}| )}{n\tau_{1}2^{2l-3}r^{2}_{n}-C_{3}\varphi _{n1}k_{n}^{1/2}\gamma2^{l}r_{n}}. \end{aligned}$$

Using the Cauchy-Schwarz inequality, we have

$$\begin{aligned} E \Bigl(\sup_{\boldsymbol {\beta }\in S_{nl}}|{{\mathrm{I}}}_{n2}| \Bigr) \leq&2\bigl[E\bigl(\bigl(\boldsymbol {f}(\mathbf{U})+\boldsymbol {\varepsilon }\bigr)^{T}(I-H)\mathbb {X}\mathbb {X}^{T}(I-H) \bigl(\boldsymbol {f}(\mathbf{U})+ \boldsymbol {\varepsilon }\bigr)\bigr) \bigr]^{1/2} \\ &{}\times \Bigl[E \Bigl(\sup_{\boldsymbol {\beta }\in S_{nl}}\|\boldsymbol {\beta }-\boldsymbol {\beta }_{0} \|^{2} \Bigr) \Bigr]^{1/2} \\ \leq&2^{l+3/2}r_{n}\bigl[E\bigl(\boldsymbol {\varepsilon }^{T}(I-H)\mathbb {X}\mathbb {X}^{T}(I-H)\boldsymbol {\varepsilon }\bigr) \\ &{}+E\bigl(\boldsymbol {f}(\mathbf{U})^{T} (I-H)\mathbb {X}\mathbb {X}^{T}(I-H) \boldsymbol {f}(\mathbf{U})\bigr)\bigr]^{1/2}, \end{aligned}$$

where

$$ E\bigl(\boldsymbol {\varepsilon }^{T}(I-H)\mathbb {X}\mathbb {X}^{T}(I-H)\boldsymbol {\varepsilon }\bigr) = \sigma^{2}E\bigl(\operatorname{tr}\bigl((I-H)\mathbb {X}\mathbb {X}^{T}(I-H) \bigr)\bigr) =O(np_{n}) $$

and

$$\begin{aligned}& E\bigl[\boldsymbol {f}(\mathbf{U})^{T} (I-H)\mathbb {X}\mathbb {X}^{T}(I-H) \boldsymbol {f}( \mathbf{U})\bigr] \\& \quad \leq E\bigl[\operatorname{tr}\bigl(\mathbb {X}^{T}(I-H)\mathbb {X}\bigr) \operatorname{tr}\bigl(\boldsymbol {f}(\mathbf{U})^{T} (I-H)\boldsymbol {f}(\mathbf{U})\bigr) \bigr] \\& \quad =O(np_{n})O\bigl(nq_{n}^{-2r}\bigr) =O \bigl(n^{2}p_{n}q_{n}^{-2r}\bigr). \end{aligned}$$

Accordingly,

$$ E \Bigl(\sup_{\boldsymbol {\beta }\in S_{nl}}|{{\mathrm{I}}}_{n2}| \Bigr)\leq C_{4}2^{l}r_{n}\bigl(\sqrt{np_{n}}+n \sqrt{p_{n}}q_{n}^{-r}\bigr). $$

Then we can get

$$ \sum_{l>L,2^{l}r_{n}\leq2^{L_{1}}\mu_{n}}\Pr(\hat {\boldsymbol {\beta }}\in S_{nl}) \leq\sum _{l>L}\frac{C_{4}2^{l}r_{n}(\sqrt{np_{n}}+n\sqrt {p_{n}}q_{n}^{-r})}{n\tau_{1}2^{2l-3}r^{2}_{n}-C_{3}\varphi_{n1}k_{n}^{1/2}\gamma2^{l}r_{n}}. $$

We choose $r_{n}=(\sqrt{p_{n}/n}+\sqrt{p_{n}}q_{n}^{-r})$, we have

$$\sum_{l>L,2^{l}r_{n}\leq2^{L_{1}}\mu_{n}}\Pr(\hat {\boldsymbol {\beta }}\in S_{nl})=\sum _{l>L}\frac{C_{4}}{\tau_{1}2^{l-3}-C_{3}\varphi _{n1}k_{n}^{1/2}\gamma/(\sqrt{np_{n}}+n\sqrt{p_{n}}q_{n}^{-r})}. $$

By condition (A5)(a) $\varphi_{n1}k_{n}^{1/2}/(\sqrt{np_{n}}+n\sqrt{p_{n}}q_{n}^{-r})\rightarrow0$, for sufficiently large *n*,

$$2^{l-3}-C_{3}\tau_{1}^{-1} \lambda_{j} k_{n}^{1/2}/\bigl(\sqrt{np_{n}}+n \sqrt{p_{n}}M_{n}^{-r_{g}}\bigr)\geq2^{l-4}. $$

Thus

$$\sum_{l>L,2^{l}r_{n}\leq2^{L_{1}}\mu_{n}}\Pr(\hat {\boldsymbol {\beta }}\in S_{nl})\leq\sum _{l>L}\frac{C_{4}}{2^{l-4}}\leq C_{4}2^{-(L-5)}. $$

Let $L\rightarrow\infty$, then

$$\sum_{l>L,2^{l}r_{n}\leq2^{L_{1}}\mu_{n}}\Pr(\hat {\boldsymbol {\beta }}\in S_{nl})\rightarrow0. $$

Hence

$$\|\hat {\boldsymbol {\beta }}-\boldsymbol {\beta }_{0}\|=O_{P}\bigl(\sqrt{n^{-1}p_{n}}+ \sqrt{p_{n}}q_{n}^{-r}\bigr). $$

 □

### Proof of Theorem 3.3

(i) By Theorem 3.2, for sufficiently large $C_{5}$, $\hat {\boldsymbol {\beta }}$ lies in the ball $\{\boldsymbol {\beta }:\|\boldsymbol {\beta }-\boldsymbol {\beta }_{0}\|\leq v_{n}C_{5}\}$ with probability converging to 1, where $v_{n}=\sqrt{n^{-1}p_{n}}+\sqrt{p_{n}}q_{n}^{-r}$. Let $\boldsymbol {\beta }_{(1)}=\boldsymbol {\beta }_{10}+v_{n}\boldsymbol {\nu }_{1}$ and $\boldsymbol {\beta }_{(2)}=\boldsymbol {\beta }_{20}+v_{n}\boldsymbol {\nu }_{2}=v_{n}\boldsymbol {\nu }_{2}$ with $\|\boldsymbol {\nu }\|^{2}=\|\boldsymbol {\nu }_{1}\|^{2}+\|\boldsymbol {\nu }_{2}\|^{2}\leq C_{5}^{2}$. Let

$$V_{n}(\boldsymbol {\nu }_{1},\boldsymbol {\nu }_{2})={Q}_{n}( \boldsymbol {\beta }_{(1)},\boldsymbol {\beta }_{(2)})-{Q}_{n}(\boldsymbol {\beta }_{10},{ \mathbf{0}})= {Q}_{n}(\boldsymbol {\beta }_{10}+v_{n} \boldsymbol {\nu }_{1},v_{n}\boldsymbol {\nu }_{2})-{Q}_{n}( \boldsymbol {\beta }_{10},{\mathbf{0}}). $$

Then $\hat {\boldsymbol {\beta }}_{1}$ and $\hat {\boldsymbol {\beta }}_{2}$ can be attained by minimizing $V_{n}(\boldsymbol {\nu }_{1},\boldsymbol {\nu }_{2})$ over $\|\boldsymbol {\nu }\|\leq C_{5}$, except on an event with probability converging to zero. We only need to show that, for some $\boldsymbol {\nu }_{1}$ and $\boldsymbol {\nu }_{2}$ with $\|\boldsymbol {\nu }\|\leq C_{5}$, if $\|\boldsymbol {\nu }_{2}\|>0$,

$$\Pr\bigl(V_{n}(\boldsymbol {\nu }_{1},\boldsymbol {\nu }_{2})-V_{n}( \boldsymbol {\nu }_{1},{\mathbf{0}})>0\bigr)\rightarrow1, \quad n\rightarrow\infty. $$

Some simple calculations show that

$$\begin{aligned} V_{n}(\boldsymbol {\nu }_{1},\boldsymbol {\nu }_{2})-V_{n}( \boldsymbol {\nu }_{1},{\mathbf{0}}) =&v_{n}^{2} \bigl\Vert (I-H)\mathbb {X}_{2}\boldsymbol {\nu }_{2} \bigr\Vert ^{2}+2v_{n}^{2}( \mathbb {X}_{1}\boldsymbol {\nu }_{1})^{T}(I-H) (\mathbb {X}_{2} \boldsymbol {\nu }_{2}) \\ &{} -2v_{n}\bigl(\boldsymbol {f}(\mathbf{U})+\boldsymbol {\varepsilon }\bigr)^{T}(I-H) ( \mathbb {X}_{2}\boldsymbol {\nu }_{2}) +\sum_{j\notin \mathcal {A}} \lambda_{j}\|v_{n}\boldsymbol {\nu }_{2j}\|^{\gamma} \\ \stackrel{\Delta}{=}&\mathrm{II}_{n1}+\mathrm{II}_{n2}+ \mathrm {II}_{n3}+\mathrm{II}_{n4}. \end{aligned}$$

For the first two terms $\mathrm{II}_{n1}$ and $\mathrm{II}_{n2}$,

$$ \mathrm{II}_{n1}+\mathrm{II}_{n2} \geq -v_{n}^{2} \bigl\Vert (I-H)\mathbb {X}_{1}\boldsymbol {\nu }_{1} \bigr\Vert ^{2} =-nv_{n}^{2}C_{5}^{2} \bigl(o_{P}(1)+\tau_{2}\bigr). $$

For $\mathrm{II}_{n3}$, we have

$$\begin{aligned}& E\bigl[\bigl(\boldsymbol {f}(\mathbf{U})+\boldsymbol {\varepsilon }\bigr)^{T}(I-H)\mathbb {X}_{2} \boldsymbol {\nu }_{2}\bigr]^{2} \\& \quad \leq2\bigl\{ E\bigl[\boldsymbol {f}(\mathbf{U})^{T}(I-H)\mathbb {X}_{2} \boldsymbol {\nu }_{2}\boldsymbol {\nu }_{2}^{T}\mathbb {X}^{T}_{2}(I-H) \boldsymbol {f}(\mathbf{U}) \\& \qquad {}+\boldsymbol {\varepsilon }^{T}(I-H)\mathbb {X}_{2}\boldsymbol {\nu }_{2} \boldsymbol {\nu }_{2}^{T}\mathbb {X}^{T}_{2}(I-H)\boldsymbol {\varepsilon }\bigr]\bigr\} \\& \quad \leq C_{6}\bigl\{ E\bigl[\operatorname{tr}\bigl( \mathbb {X}^{T}_{2}(I-H)\mathbb {X}_{2}\bigr)\operatorname{tr} \bigl(\boldsymbol {f}(\mathbf{U})^{T}(I-H)\boldsymbol {f}(\mathbf{U})\bigr)\bigr] \\& \qquad {}+\sigma^{2}E\bigl[\operatorname{tr}\bigl( \mathbb {X}^{T}_{2}(I-H)\mathbb {X}_{2}\bigr)\bigr]\bigr\} \\& \quad =O\bigl(n^{2}p_{n}q_{n}^{-2r}+np_{n} \bigr). \end{aligned}$$

Thus we have

$$\mathrm{II}_{n3}=v_{n}\bigl(np_{n}^{1/2}q_{n}^{-r}+n^{1/2}p_{n}^{1/2} \bigr)O_{P}(1). $$

For $\mathrm{II}_{n4}$, by $0<\gamma<1$,

$$\biggl(\sum_{j\notin \mathcal {A}} \|v_{n} \boldsymbol {\nu }_{2j}\|^{\gamma} \biggr)^{1/\gamma }\geq \biggl(\sum _{j\notin \mathcal {A}}\|v_{n}\boldsymbol {\nu }_{2j} \|^{2} \biggr)^{1/2}=v_{n}\| \boldsymbol {\nu }_{2}\|. $$

Accordingly,

$$\mathrm{II}_{n4}\geq\varphi_{n2}v_{n}^{\gamma} \|\boldsymbol {\nu }_{2}\|^{\gamma}. $$

By condition (A5)(b), for some $\|\boldsymbol {\nu }_{2}\|>0$, we have

$$\Pr\bigl(V_{n}(\boldsymbol {\nu }_{1},\boldsymbol {\nu }_{2})-V_{n}( \boldsymbol {\nu }_{1},{\mathbf{0}})>0\bigr)\rightarrow1. $$

(ii) Let $\boldsymbol {\omega }_{n}$ be some $\sum_{j=1}^{k_{n}}d_{j}$-vector with $\|\boldsymbol {\omega }_{n}\| ^{2}=1$. By Theorem 3.2(i), with probability tending to 1, we have the following result:

$$\frac{\partial{Q}_{n}(\boldsymbol {\beta }_{(1)})}{\partial \boldsymbol {\beta }_{(1)}} \bigg|_{\boldsymbol {\beta }_{(1)}=\hat {\boldsymbol {\beta }}_{(1)}}=\mathbb {X}^{T}_{1}(I-H) \mathbb {X}_{1}(\hat {\boldsymbol {\beta }}_{(1)}-\boldsymbol {\beta }_{10}) -\mathbb {X}^{T}_{1}(I-H) \bigl(\boldsymbol {f}(\mathbf{U})+\boldsymbol {\varepsilon }\bigr)+{\boldsymbol{\xi}}_{n}={\mathbf{0}}, $$

where ${\boldsymbol{\xi}}_{n}=(\lambda_{1}\gamma\|\hat {\boldsymbol {\beta }}_{1}\|^{\gamma-2}\hat {\boldsymbol {\beta }}_{1}^{T},\ldots,\lambda_{k_{n}}\gamma\|\hat {\boldsymbol {\beta }}_{k_{n}}\|^{\gamma-2}\hat {\boldsymbol {\beta }}_{k_{n}}^{T})^{T}$. We consider the limit distribution

$$\begin{aligned}& n^{-1/2}\boldsymbol {\omega }_{n}^{T}\Omega^{-1/2}_{11} \bigl[\mathbb {X}^{T}_{1}(I-H)\mathbb {X}_{1}\bigr](\hat {\boldsymbol {\beta }}_{(1)}-\boldsymbol {\beta }_{10}) \\& \quad = n^{-1/2}\boldsymbol {\omega }_{n}^{T}\Omega^{-1/2}_{11} \mathbb {X}^{T}_{1}(I-H)\boldsymbol {f}(\mathbf {U})+n^{-1/2} \boldsymbol {\omega }_{n}^{T}\Omega^{-1/2}_{11} \mathbb {X}^{T}_{1}(I-H)\boldsymbol {\varepsilon }\\& \qquad {}-n^{-1/2}\boldsymbol {\omega }_{n}^{T} \Omega^{-1/2}_{11}{\boldsymbol{\xi}}_{n} \\& \quad \stackrel{\Delta}{=} J_{n1}+J_{n2}+J_{n3}. \end{aligned}$$

For $J_{n1}$,

$$ J_{n1}^{2} = n^{-1} \bigl\vert \boldsymbol {\omega }_{n}^{T}\Omega^{-1/2}_{11} \mathbb {X}^{T}_{1}(I-H)\boldsymbol {f}(\mathbf{U}) \bigr\vert ^{2} =O_{P}\bigl(nq_{n}^{-2r}\bigr). $$

For $J_{n3}$, by conditions (A2) and (A4), we have

$$ E\bigl(J_{n3}^{2}\bigr) \leq n^{-1} \tau^{-1}_{1}\varphi_{n1}\gamma^{2}\sum _{j=1}^{k_{n}}E\|\hat {\boldsymbol {\beta }}_{j} \|^{2(\gamma-1)} =O\bigl(n^{-1}\varphi_{n1}k_{n} \bigr). $$

For $J_{n2}$,

$$\begin{aligned} J_{n2} =&n^{-1/2}\boldsymbol {\omega }_{n}^{T} \Omega^{-1/2}_{11}\mathbf {G}^{T}_{1}(I-H)\boldsymbol {\varepsilon }+n^{-1/2}\boldsymbol {\omega }_{n}^{T}\Omega^{-1/2}_{11} \widetilde {\mathbb {X}}^{T}_{1}\boldsymbol {\varepsilon }\\ &{}-n^{-1/2}\boldsymbol {\omega }_{n}^{T} \Omega^{-1/2}_{11}\widetilde {\mathbb {X}}^{T}_{1}H\boldsymbol {\varepsilon }\\ \stackrel{\Delta}{=}&K_{n1}+K_{n2}+K_{n3}. \end{aligned}$$

Under conditions (C1) and (C2),

$$\begin{aligned} EK_{n1}^{2} =&n^{-1}\boldsymbol {\omega }_{n}^{T} \Omega^{-1/2}_{11}E\bigl[\mathbf {G}^{T}_{1}(I-H) \boldsymbol {\varepsilon }\boldsymbol {\varepsilon }^{T}(I-H)\mathbf {G}_{1}\bigr]\Omega^{-1/2}_{11} \boldsymbol {\omega }_{n} \\ =&O\bigl(k_{n}q_{n}^{-2r}\bigr). \end{aligned}$$

By condition (A6), we have

$$\begin{aligned} EK_{n3}^{2} =&n^{-1}\boldsymbol {\omega }_{n}^{T} \Omega^{-1/2}_{11}E\bigl[\widetilde {\mathbb {X}}^{T}_{1}H\boldsymbol {\varepsilon }\boldsymbol {\varepsilon }^{T}H\widetilde {\mathbb {X}}_{1}\bigr]\Omega^{-1/2}_{11} \boldsymbol {\omega }_{n} \\ =& O\bigl(n^{-1}k_{n}q_{n}\bigr). \end{aligned}$$

Now we focus on $K_{n2}$

$$K_{n2}=n^{-1/2}\boldsymbol {\omega }_{n}^{T} \Omega^{-1/2}_{11}\widetilde {\mathbb {X}}^{T}_{1}\boldsymbol {\varepsilon }\stackrel {\Delta}{=}\frac{1}{\sqrt{n}}\sum_{i=1}^{n}s_{ni} \varepsilon_{i}. $$

First,

$$\begin{aligned}& E(s_{ni}\varepsilon_{i}) = 0; \\& \operatorname{Var} \Biggl(\sum_{i=1}^{n}s_{ni} \varepsilon_{i} \Biggr) = \sum_{i=1}^{n} \operatorname{Var}(s_{ni}\varepsilon_{i})= \sigma^{2}. \end{aligned}$$

Next we verify the conditions of the Lindeberg-Feller central limit. For any $\epsilon>0$,

$$\begin{aligned} \sum_{i=1}^{n}E\bigl[\bigl(s_{ni}^{2} \varepsilon_{i}^{2}\bigr)\mathbf {1}\bigl( \vert s_{ni}\varepsilon_{i} \vert >\epsilon\bigr)\bigr] =&nE \bigl[\bigl(s_{n1}^{2}\varepsilon_{1}^{2} \bigr)\mathbf{1}\bigl( \vert s_{n1}\varepsilon _{1} \vert >\epsilon\bigr)\bigr] \\ \leq&n\bigl[E\bigl(s_{n1}^{4}\varepsilon_{1}^{4} \bigr)\bigr]^{1/2}\bigl[\Pr\bigl( \vert s_{n1}\varepsilon _{1} \vert >\epsilon\bigr)\bigr]^{1/2} . \end{aligned}$$

By condition (A6),

$$\begin{aligned} E\bigl(s_{n1}^{4}\varepsilon_{1}^{4} \bigr) =&n^{-2}E\bigl\{ \boldsymbol {\omega }_{n}^{T} \Omega^{-1/2}_{11}\bigl[\mathbf {x}_{1}-E( \mathbf {x}_{1}|U_{1})\bigr] \bigl[\mathbf {x}_{1}-E( \mathbf {x}_{1}|U_{1})\bigr]^{T}\Omega^{-1/2}_{11} \boldsymbol {\omega }_{n}\bigr\} ^{2} E\varepsilon_{1}^{4} \\ \leq& n^{-2}\rho_{\min}^{2}\bigl(\boldsymbol {\omega }_{n} \boldsymbol {\omega }_{n}^{T}\bigr)\rho_{\max}^{2}\bigl(\Omega ^{-1}_{11}\bigr)E\bigl\{ \bigl[\mathbf {x}_{1}-E( \mathbf {x}_{1}|U_{1})\bigr]^{T}\bigl[ \mathbf {x}_{1}-E(\mathbf {x}_{1}|U_{1})\bigr]\bigr\} ^{2} E\varepsilon_{1}^{4} \\ \leq& n^{-2}\rho_{\min}^{2}\bigl(\boldsymbol {\omega }_{n} \boldsymbol {\omega }_{n}^{T}\bigr)\rho_{\max}^{2}\bigl(\Omega ^{-1}_{11}\bigr)E\varepsilon_{1}^{4} k_{n}d^{*} \sum_{j=1}^{k_{n}}\sum _{k=1}^{d_{j}}E\bigl[X_{1jk}-E(X_{1jk}|U_{1}) \bigr]^{4} \\ =&O\bigl(k_{n}^{2}n^{-2}\bigr) \end{aligned}$$

and

$$\begin{aligned} P\bigl( \vert s_{n1}\varepsilon_{1} \vert >\epsilon \bigr) \leq& \frac{1}{\epsilon ^{2}}E(s_{n1}\varepsilon_{1})^{2} \\ =&\frac{\sigma^{2}}{\epsilon^{2}}n^{-1}\boldsymbol {\omega }_{n}^{T} \Omega^{-1/2}_{11}E\bigl\{ \bigl[\mathbf {x}_{1}-E( \mathbf {x}_{1}|U_{1})\bigr] \bigl[\mathbf {x}_{1}-E( \mathbf {x}_{1}|U_{1})\bigr]^{T}\bigr\} \Omega ^{-1/2}_{11}\boldsymbol {\omega }_{n} \\ =&\frac{\sigma^{2}}{\epsilon^{2}}n^{-1}=O\bigl(n^{-1}\bigr). \end{aligned}$$

Thus we have

$$\sum_{i=1}^{n}E\bigl[\bigl(s_{ni}^{2} \varepsilon_{i}^{2}\bigr)\mathbf {1}\bigl( \vert s_{ni}\varepsilon_{i} \vert >\epsilon\bigr)\bigr]=O \bigl(nk_{n}n^{-1}n^{-1/2}\bigr)=o(1). $$

This means that $K_{n2}\stackrel{D}{\rightarrow}N(0,\sigma^{2})$. Using Slutsky’s theorem, we have

$$n^{-1/2}\boldsymbol {\omega }_{n}^{T}\Omega^{-1/2}_{11} \bigl[\mathbb {X}^{T}_{1}(I-H)\mathbb {X}_{1}\bigr](\hat {\boldsymbol {\beta }}_{(1)}-\boldsymbol {\beta }_{10})\stackrel{D}{\rightarrow}N\bigl(0, \sigma^{2}\bigr). $$

Let $u_{n}^{2}=n^{2}\boldsymbol {\omega }_{n}^{T}(\mathbb {X}^{T}_{1}(I-H)\mathbb {X}_{1})^{-1}\Omega_{11}(\mathbb {X}^{T}_{1}(I-H)\mathbb {X}_{1})^{-1}\boldsymbol {\omega }_{n}$, then

$$n^{1/2}u_{n}^{-1}\boldsymbol {\omega }_{n}^{T}( \hat {\boldsymbol {\beta }}_{(1)}-\boldsymbol {\beta }_{10})\stackrel{D}{\rightarrow }N\bigl(0, \sigma^{2}\bigr). $$

 □
